# 2,7-Dichloro-4-(chloro­acet­yl)fluorene

**DOI:** 10.1107/S1600536809027287

**Published:** 2009-07-29

**Authors:** Jian-Bo Chu, Guo-Wu Rao, Jin-Hao Zhao

**Affiliations:** a Department of Pharmacy, Zhejiang Medical College, Hangzhou 310053, People’s Republic of China; b College of Pharmaceutical Science, Zhejiang University of Technology, Hangzhou,310032, People’s Republic of China; c College of Agriculture and Biotechnology, Zhejiang University, Hangzhou 310029, People’s Republic of China

## Abstract

There are two mol­ecules in the asymmetric unit of the title compound, C_15_H_9_Cl_3_O. The fluorene rings of the two mol­ecules are both coplanar within 066 (3) Å. In the crystal, C—H⋯O and C—H⋯Cl hydrogen bonds link the mol­ecules into sheets running parallel to (100).

## Related literature

The title compound is an important inter­mediate in the synthesis of benflumetol, see: Deng *et al.* (2000 ▶). Benflumetol conforms structurally and in mode of action to the structure and mode of action of the aryl amino alcohol group of anti­malarial drugs, including quinine, mefloquine, and halofantrine, see: Pradines *et al.* (1999 ▶). For our ongoing work on structure–activity relationships, see: Rao & Hu (2005 ▶, 2006 ▶); Hu *et al.* (2004 ▶).
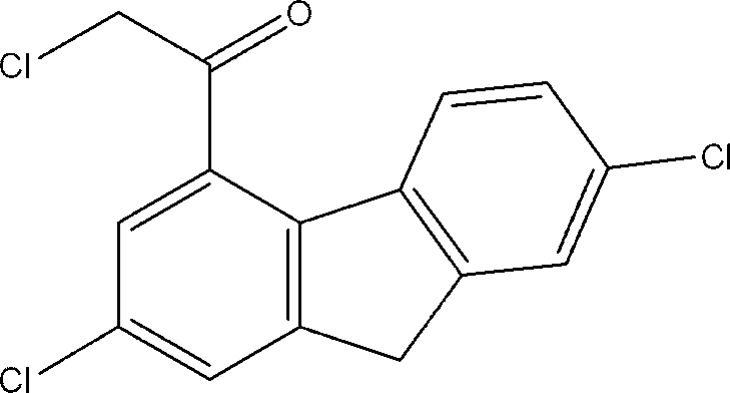


## Experimental

### 

#### Crystal data

C_15_H_9_Cl_3_O
*M* *_r_* = 311.57
Triclinic, 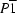

*a* = 7.607 (6) Å
*b* = 13.227 (10) Å
*c* = 14.957 (11) Å
α = 64.942 (9)°
β = 81.653 (10)°
γ = 76.433 (10)°
*V* = 1323.5 (17) Å^3^
*Z* = 4
Mo *K*α radiation
μ = 0.68 mm^−1^
*T* = 298 K
0.25 × 0.15 × 0.10 mm

#### Data collection

Bruker SMART CCD area detector diffractometer
Absorption correction: multi-scan (*ABSCOR*; Higashi, 1995 ▶) *T*_min_ = 0.849, *T*_max_ = 0.935
6098 measured reflections
5096 independent reflections
3419 reflections with *I* > 2σ(*I*)
*R*_int_ = 0.035

#### Refinement

*R*[*F* ^2^ > 2σ(*F* ^2^)] = 0.064
*wR*(*F* ^2^) = 0.174
*S* = 0.96
5096 reflections
343 parameters
H-atom parameters constrained
Δρ_max_ = 0.53 e Å^−3^
Δρ_min_ = −0.44 e Å^−3^

### 

Data collection: *CAD-4 EXPRESS* (Enraf–Nonius, 1994 ▶); cell refinement: *CAD-4 EXPRESS*; data reduction: *XCAD4* (Harms & Wocadlo, 1995 ▶); program(s) used to solve structure: *SHELXS97* (Sheldrick, 2008 ▶); program(s) used to refine structure: *SHELXL97* (Sheldrick, 2008 ▶); molecular graphics: *ORTEP-3 for Windows* (Farrugia, 1997 ▶); software used to prepare material for publication: *WinGX* (Farrugia, 1999 ▶).

## Supplementary Material

Crystal structure: contains datablocks I, global. DOI: 10.1107/S1600536809027287/kp2225sup1.cif

Structure factors: contains datablocks I. DOI: 10.1107/S1600536809027287/kp2225Isup2.hkl

Additional supplementary materials:  crystallographic information; 3D view; checkCIF report

## Figures and Tables

**Table 1:** Hydrogen-bond geometry (Å, °)

*D*—H⋯*A*	*D*—H	H⋯*A*	*D*⋯*A*	*D*—H⋯*A*
C1—H1⋯O1	0.93	2.37	2.998 (4)	124
C15—H15*B*⋯Cl6	0.97	2.80	3.678 (5)	151
C21—H21⋯O2	0.93	2.45	3.086 (5)	126
C35—H35*A*⋯O1^i^	0.97	2.45	3.261 (5)	140

Symmetry code: (i) 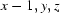.

**Fig. 1.**
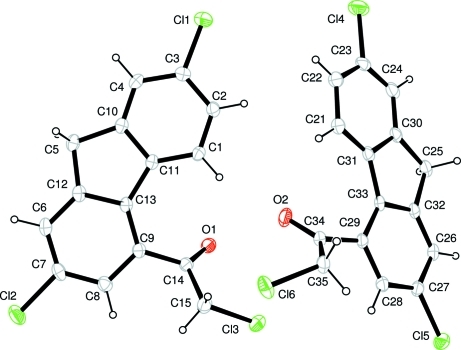
The structure of (I) showing the atom-labelling scheme. Ellipsoids are drawn at the 30% probability level.

**Fig. 2.**
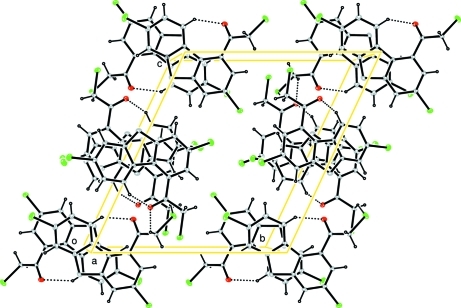
The crystal packing of (I) showing the C—H···O and C—H···Cl hydrogen bonds.

## supplementary crystallographic information

### Comment

Benflumetol is a racemic fluorene derivative. It conforms to the structure and reactivity of the aryl amino alcohol group of antimalarial drugs, including quinine, mefloquine, and halofantrine (Pradines *et al.*, 1999). 2,7-Dichloro-4-chloroacetyl fluorene, (I), is an important intermediate in the synthesis of benflumetol (Deng *et al.*, 2000). In a continuation of our work on the structure-activity relationships (Rao *et al.*, 2004, 2005, 2006) we have obtained a colourless crystalline compound as the product of the reaction of 2-chloroacetyl chloride and 2,7-dichloro-9*H*-fluorene. The structural characterization of our product, (I), was performed by single-crystal X-ray diffraction.

The two essentially identical molecules form the asymmetric unit of (I) (Fig. 1). The two independent molecules are roughly parallel to each other, with a head-to-head orientation. The molecular structure is built up from three fused ring, two of which are six-membered and one five-membered. In both molecules, the three rings in the fluorene are coplanar with the largest deviation from the planes being 0.0664 (27) Å for atom C9 and 0.0656 (28)Å for atom C29, respectively. In both molecules, the dihedral angle between the two planes of the fluorene ring is 35.59 (6)°. The torsion angles for substituted COCH_2_Cl (O1/C14/C15/Cl3 and O2/C34/C35/Cl6) are -8.4 (5) and 31.8 (5)*^o^*, respectively.

The crystal packing of (I) is defined by C—H···O and C—H···Cl hydrogen bonds that link the molecules into sheets running parallel to the (100) plane (Table 1, Fig. 2).

### Experimental

The title compound was prepared from 2-chloroacetyl chloride and 2,7-dichloro-9*H*-fluorene according to the procedure of Deng *et al.* (2000). A solution of the compound in ethanol was concentrated gradually at room temperature to afford colourless prisms (m.p. 398–399 K).

### Refinement

H atoms were included in calculated positions and refined using a riding model. H atoms were given isotropic displacement parameters equal to 1.2 times the equivalent isotropic displacement parameters of their parent atoms and C—H distances were restrained to 0.97 Å for methylene H atoms, 0.93 Å for aromatic H atoms.

### Crystal data

**Table:**

C_15_H_9_Cl_3_O	*Z* = 4
*M**_r_* = 311.57	*F*(000) = 632
Triclinic, *P*1	*D*_x_ = 1.564 Mg m^−^^3^
Hall symbol: -P 1	Melting point = 398–399 K
*a* = 7.607 (6) Å	Mo *K*α radiation, λ = 0.71073 Å
*b* = 13.227 (10) Å	Cell parameters from 868 reflections
*c* = 14.957 (11) Å	θ = 2.7–23.5°
α = 64.942 (9)°	µ = 0.68 mm^−^^1^
β = 81.653 (10)°	*T* = 298 K
γ = 76.433 (10)°	Prismatic, colorless
*V* = 1323.5 (17) Å^3^	0.25 × 0.15 × 0.10 mm

### Data collection

**Table:**

Bruker SMART CCD area detector diffractometer	5096 independent reflections
Radiation source: fine-focus sealed tube	3419 reflections with *I* > 2σ(*I*)
graphite	*R*_int_ = 0.035
φ and ω scans	θ_max_ = 26.0°, θ_min_ = 1.5°
Absorption correction: multi-scan (*ABSCOR*; Higashi, 1995)	*h* = −9→9
*T*_min_ = 0.849, *T*_max_ = 0.935	*k* = −16→14
6098 measured reflections	*l* = −17→18

### Refinement

**Table:**

Refinement on *F*^2^	Primary atom site location: structure-invariant direct methods
Least-squares matrix: full	Secondary atom site location: difference Fourier map
*R*[*F*^2^ > 2σ(*F*^2^)] = 0.064	Hydrogen site location: inferred from neighbouring sites
*wR*(*F*^2^) = 0.174	H-atom parameters constrained
*S* = 0.96	*w* = 1/[σ^2^(*F*_o_^2^) + (0.1131*P*)^2^] where *P* = (*F*_o_^2^ + 2*F*_c_^2^)/3
5096 reflections	(Δ/σ)_max_ < 0.001
343 parameters	Δρ_max_ = 0.53 e Å^−^^3^
0 restraints	Δρ_min_ = −0.44 e Å^−^^3^

### Special details

**Table:**

Geometry. All e.s.d.'s (except the e.s.d. in the dihedral angle between two l.s. planes) are estimated using the full covariance matrix. The cell e.s.d.'s are taken into account individually in the estimation of e.s.d.'s in distances, angles and torsion angles; correlations between e.s.d.'s in cell parameters are only used when they are defined by crystal symmetry. An approximate (isotropic) treatment of cell e.s.d.'s is used for estimating e.s.d.'s involving l.s. planes.
Refinement. Refinement of *F*^2^ against ALL reflections. The weighted *R*-factor *wR* and goodness of fit *S* are based on *F*^2^, conventional *R*-factors *R* are based on *F*, with *F* set to zero for negative *F*^2^. The threshold expression of *F*^2^ > σ(*F*^2^) is used only for calculating *R*-factors(gt) *etc*. and is not relevant to the choice of reflections for refinement. *R*-factors based on *F*^2^ are statistically about twice as large as those based on *F*, and *R*- factors based on ALL data will be even larger.

### Fractional atomic coordinates and isotropic or equivalent isotropic displacement parameters (Å^2^)

**Table:**

	*x*	*y*	*z*	*U*_iso_*/*U*_eq_	
Cl1	0.38813 (15)	1.35826 (8)	0.52879 (8)	0.0682 (3)	
Cl2	1.12004 (16)	0.64244 (10)	0.44936 (9)	0.0768 (4)	
Cl3	0.74877 (16)	0.60526 (8)	0.91826 (7)	0.0685 (3)	
O1	0.8430 (4)	0.81774 (19)	0.77151 (16)	0.0514 (6)	
C1	0.5934 (5)	1.0272 (3)	0.6453 (2)	0.0436 (8)	
H1	0.5919	0.9673	0.7072	0.052*	
C2	0.5018 (5)	1.1346 (3)	0.6318 (2)	0.0462 (8)	
H2	0.4382	1.1474	0.6853	0.055*	
C3	0.5023 (5)	1.2242 (3)	0.5401 (2)	0.0469 (8)	
C4	0.5926 (5)	1.2073 (3)	0.4590 (2)	0.0468 (8)	
H4	0.5919	1.2675	0.3971	0.056*	
C5	0.7849 (5)	1.0590 (3)	0.3956 (2)	0.0462 (8)	
H5A	0.7058	1.0706	0.3453	0.055*	
H5B	0.8851	1.0978	0.3641	0.055*	
C6	0.9522 (5)	0.8548 (3)	0.4220 (3)	0.0503 (9)	
H6	0.9899	0.8749	0.3557	0.060*	
C7	0.9956 (5)	0.7454 (3)	0.4890 (3)	0.0530 (9)	
C8	0.9454 (5)	0.7127 (3)	0.5885 (3)	0.0489 (8)	
H8	0.9759	0.6370	0.6322	0.059*	
C9	0.8482 (4)	0.7941 (3)	0.6235 (2)	0.0408 (7)	
C10	0.6833 (4)	1.0998 (3)	0.4718 (2)	0.0403 (7)	
C11	0.6891 (4)	1.0092 (3)	0.5647 (2)	0.0381 (7)	
C12	0.8510 (4)	0.9354 (3)	0.4544 (2)	0.0436 (8)	
C13	0.7963 (4)	0.9061 (3)	0.5552 (2)	0.0384 (7)	
C14	0.8187 (4)	0.7600 (3)	0.7319 (2)	0.0419 (7)	
C15	0.7535 (6)	0.6491 (3)	0.7895 (3)	0.0588 (10)	
H15A	0.8323	0.5903	0.7719	0.071*	
H15B	0.6327	0.6574	0.7704	0.071*	
Cl4	0.11626 (18)	1.38515 (8)	0.82118 (10)	0.0877 (4)	
Cl5	0.41632 (16)	0.54224 (8)	1.28292 (6)	0.0645 (3)	
Cl6	0.26609 (15)	0.64588 (9)	0.81998 (8)	0.0681 (3)	
O2	0.3758 (4)	0.8383 (2)	0.84044 (17)	0.0593 (7)	
C21	0.1871 (5)	1.0602 (3)	0.8631 (3)	0.0507 (8)	
H21	0.1806	1.0196	0.8263	0.061*	
C22	0.1468 (5)	1.1780 (3)	0.8212 (3)	0.0566 (9)	
H22	0.1113	1.2170	0.7564	0.068*	
C23	0.1596 (5)	1.2369 (3)	0.8761 (3)	0.0534 (9)	
C24	0.2074 (5)	1.1838 (3)	0.9724 (3)	0.0553 (9)	
H24	0.2157	1.2255	1.0079	0.066*	
C25	0.2907 (5)	0.9899 (3)	1.1187 (3)	0.0512 (9)	
H25A	0.1947	1.0019	1.1656	0.061*	
H25B	0.4023	1.0013	1.1339	0.061*	
C26	0.3527 (5)	0.7703 (3)	1.1986 (2)	0.0467 (8)	
H26	0.3686	0.7666	1.2605	0.056*	
C27	0.3686 (5)	0.6728 (3)	1.1837 (2)	0.0466 (8)	
C28	0.3466 (5)	0.6773 (3)	1.0921 (2)	0.0448 (8)	
H28	0.3586	0.6101	1.0838	0.054*	
C29	0.3065 (4)	0.7813 (3)	1.0120 (2)	0.0404 (7)	
C30	0.2427 (5)	1.0668 (3)	1.0149 (3)	0.0461 (8)	
C31	0.2373 (4)	1.0032 (3)	0.9603 (2)	0.0417 (7)	
C32	0.3128 (4)	0.8733 (3)	1.1198 (2)	0.0431 (8)	
C33	0.2860 (4)	0.8817 (3)	1.0262 (2)	0.0400 (7)	
C34	0.3009 (5)	0.7782 (3)	0.9140 (2)	0.0432 (8)	
C35	0.1889 (5)	0.6998 (3)	0.9112 (2)	0.0503 (9)	
H35A	0.0648	0.7405	0.8994	0.060*	
H35B	0.1890	0.6364	0.9754	0.060*	

### Atomic displacement parameters (Å^2^)

**Table:**

	*U*^11^	*U*^22^	*U*^33^	*U*^12^	*U*^13^	*U*^23^
Cl1	0.0712 (7)	0.0497 (5)	0.0737 (7)	0.0006 (5)	−0.0030 (5)	−0.0222 (5)
Cl2	0.0728 (8)	0.0785 (7)	0.0839 (8)	−0.0045 (6)	0.0218 (6)	−0.0507 (6)
Cl3	0.0938 (9)	0.0535 (5)	0.0483 (5)	−0.0204 (5)	0.0077 (5)	−0.0116 (4)
O1	0.0671 (18)	0.0449 (13)	0.0426 (13)	−0.0150 (12)	−0.0028 (11)	−0.0162 (11)
C1	0.045 (2)	0.0457 (18)	0.0358 (17)	−0.0127 (15)	0.0024 (14)	−0.0115 (14)
C2	0.043 (2)	0.051 (2)	0.0451 (19)	−0.0108 (15)	0.0053 (15)	−0.0218 (16)
C3	0.044 (2)	0.0435 (18)	0.054 (2)	−0.0081 (15)	−0.0047 (16)	−0.0193 (16)
C4	0.050 (2)	0.0472 (19)	0.0362 (17)	−0.0163 (16)	−0.0021 (15)	−0.0066 (14)
C5	0.043 (2)	0.060 (2)	0.0384 (17)	−0.0213 (16)	0.0038 (14)	−0.0190 (15)
C6	0.047 (2)	0.068 (2)	0.0434 (19)	−0.0190 (17)	0.0103 (15)	−0.0300 (18)
C7	0.039 (2)	0.069 (2)	0.062 (2)	−0.0100 (17)	0.0099 (16)	−0.041 (2)
C8	0.044 (2)	0.0499 (19)	0.055 (2)	−0.0104 (16)	0.0046 (16)	−0.0243 (16)
C9	0.0338 (18)	0.0481 (18)	0.0427 (17)	−0.0126 (14)	0.0025 (13)	−0.0194 (14)
C10	0.0360 (18)	0.0502 (18)	0.0377 (17)	−0.0173 (15)	0.0014 (13)	−0.0171 (14)
C11	0.0341 (18)	0.0473 (17)	0.0363 (16)	−0.0149 (14)	0.0004 (13)	−0.0171 (14)
C12	0.0333 (19)	0.060 (2)	0.0445 (18)	−0.0183 (15)	0.0038 (14)	−0.0246 (16)
C13	0.0339 (18)	0.0483 (18)	0.0381 (16)	−0.0161 (14)	0.0034 (13)	−0.0198 (14)
C14	0.0335 (18)	0.0433 (17)	0.0435 (18)	−0.0041 (14)	−0.0001 (14)	−0.0148 (15)
C15	0.074 (3)	0.058 (2)	0.048 (2)	−0.026 (2)	0.0118 (18)	−0.0220 (17)
Cl4	0.0814 (9)	0.0455 (6)	0.1137 (10)	−0.0057 (5)	0.0130 (7)	−0.0201 (6)
Cl5	0.0920 (8)	0.0550 (5)	0.0389 (5)	−0.0111 (5)	−0.0018 (5)	−0.0140 (4)
Cl6	0.0670 (7)	0.0870 (7)	0.0757 (7)	−0.0203 (5)	0.0155 (5)	−0.0601 (6)
O2	0.081 (2)	0.0623 (15)	0.0380 (13)	−0.0298 (14)	0.0168 (12)	−0.0212 (12)
C21	0.045 (2)	0.055 (2)	0.049 (2)	−0.0091 (16)	0.0083 (15)	−0.0218 (17)
C22	0.046 (2)	0.054 (2)	0.058 (2)	−0.0065 (17)	0.0090 (17)	−0.0156 (18)
C23	0.035 (2)	0.0398 (18)	0.075 (3)	−0.0065 (14)	0.0175 (17)	−0.0202 (18)
C24	0.045 (2)	0.054 (2)	0.070 (3)	−0.0129 (17)	0.0195 (18)	−0.0344 (19)
C25	0.051 (2)	0.060 (2)	0.053 (2)	−0.0159 (17)	0.0136 (16)	−0.0355 (17)
C26	0.046 (2)	0.063 (2)	0.0339 (17)	−0.0135 (17)	0.0067 (14)	−0.0238 (16)
C27	0.049 (2)	0.0513 (19)	0.0378 (18)	−0.0147 (16)	0.0075 (14)	−0.0167 (15)
C28	0.049 (2)	0.0480 (18)	0.0399 (18)	−0.0103 (15)	0.0048 (14)	−0.0222 (15)
C29	0.0381 (19)	0.0473 (17)	0.0365 (16)	−0.0106 (14)	0.0091 (13)	−0.0198 (14)
C30	0.0359 (19)	0.0514 (19)	0.055 (2)	−0.0129 (15)	0.0160 (15)	−0.0285 (16)
C31	0.0313 (18)	0.0479 (18)	0.0475 (19)	−0.0103 (14)	0.0116 (14)	−0.0239 (15)
C32	0.0358 (19)	0.0544 (19)	0.0451 (18)	−0.0145 (15)	0.0143 (14)	−0.0279 (16)
C33	0.0318 (18)	0.0523 (18)	0.0374 (17)	−0.0116 (14)	0.0113 (13)	−0.0220 (14)
C34	0.044 (2)	0.0453 (17)	0.0377 (17)	−0.0058 (15)	0.0055 (14)	−0.0181 (14)
C35	0.057 (2)	0.061 (2)	0.0386 (18)	−0.0169 (18)	0.0086 (15)	−0.0269 (16)

### Geometric parameters (Å, °)

**Table:**

Cl1—C3	1.730 (4)	Cl4—C23	1.744 (4)
Cl2—C7	1.737 (4)	Cl5—C27	1.735 (3)
Cl3—C15	1.758 (4)	Cl6—C35	1.764 (3)
O1—C14	1.203 (4)	O2—C34	1.207 (4)
C1—C2	1.371 (5)	C21—C22	1.385 (5)
C1—C11	1.395 (5)	C21—C31	1.388 (5)
C1—H1	0.9300	C21—H21	0.9300
C2—C3	1.382 (4)	C22—C23	1.375 (5)
C2—H2	0.9300	C22—H22	0.9300
C3—C4	1.383 (5)	C23—C24	1.369 (5)
C4—C10	1.372 (5)	C24—C30	1.375 (5)
C4—H4	0.9300	C24—H24	0.9300
C5—C12	1.491 (5)	C25—C30	1.494 (5)
C5—C10	1.503 (5)	C25—C32	1.504 (5)
C5—H5A	0.9700	C25—H25A	0.9700
C5—H5B	0.9700	C25—H25B	0.9700
C6—C7	1.361 (5)	C26—C27	1.374 (5)
C6—C12	1.382 (5)	C26—C32	1.375 (5)
C6—H6	0.9300	C26—H26	0.9300
C7—C8	1.384 (5)	C27—C28	1.379 (5)
C8—C9	1.403 (5)	C28—C29	1.391 (4)
C8—H8	0.9300	C28—H28	0.9300
C9—C13	1.397 (4)	C29—C33	1.401 (4)
C9—C14	1.486 (5)	C29—C34	1.491 (4)
C10—C11	1.396 (4)	C30—C31	1.409 (4)
C11—C13	1.468 (5)	C31—C33	1.475 (5)
C12—C13	1.412 (4)	C32—C33	1.398 (5)
C14—C15	1.512 (5)	C34—C35	1.505 (5)
C15—H15A	0.9700	C35—H35A	0.9700
C15—H15B	0.9700	C35—H35B	0.9700
			
C2—C1—C11	118.9 (3)	C22—C21—C31	119.6 (3)
C2—C1—H1	120.5	C22—C21—H21	120.2
C11—C1—H1	120.5	C31—C21—H21	120.2
C1—C2—C3	121.0 (3)	C23—C22—C21	119.5 (4)
C1—C2—H2	119.5	C23—C22—H22	120.3
C3—C2—H2	119.5	C21—C22—H22	120.3
C2—C3—C4	120.8 (3)	C24—C23—C22	122.7 (3)
C2—C3—Cl1	118.7 (3)	C24—C23—Cl4	118.3 (3)
C4—C3—Cl1	120.5 (3)	C22—C23—Cl4	119.1 (3)
C10—C4—C3	118.4 (3)	C23—C24—C30	117.9 (3)
C10—C4—H4	120.8	C23—C24—H24	121.0
C3—C4—H4	120.8	C30—C24—H24	121.0
C12—C5—C10	103.2 (3)	C30—C25—C32	102.9 (3)
C12—C5—H5A	111.1	C30—C25—H25A	111.2
C10—C5—H5A	111.1	C32—C25—H25A	111.2
C12—C5—H5B	111.1	C30—C25—H25B	111.2
C10—C5—H5B	111.1	C32—C25—H25B	111.2
H5A—C5—H5B	109.1	H25A—C25—H25B	109.1
C7—C6—C12	118.6 (3)	C27—C26—C32	118.3 (3)
C7—C6—H6	120.7	C27—C26—H26	120.9
C12—C6—H6	120.7	C32—C26—H26	120.9
C6—C7—C8	122.3 (3)	C26—C27—C28	121.3 (3)
C6—C7—Cl2	119.3 (3)	C26—C27—Cl5	118.8 (3)
C8—C7—Cl2	118.4 (3)	C28—C27—Cl5	119.9 (3)
C7—C8—C9	119.9 (3)	C27—C28—C29	120.6 (3)
C7—C8—H8	120.0	C27—C28—H28	119.7
C9—C8—H8	120.0	C29—C28—H28	119.7
C13—C9—C8	118.6 (3)	C28—C29—C33	118.9 (3)
C13—C9—C14	122.6 (3)	C28—C29—C34	117.0 (3)
C8—C9—C14	118.6 (3)	C33—C29—C34	124.0 (3)
C4—C10—C11	121.4 (3)	C24—C30—C31	121.3 (3)
C4—C10—C5	128.6 (3)	C24—C30—C25	128.1 (3)
C11—C10—C5	110.0 (3)	C31—C30—C25	110.6 (3)
C1—C11—C10	119.3 (3)	C21—C31—C30	118.9 (3)
C1—C11—C13	131.7 (3)	C21—C31—C33	133.4 (3)
C10—C11—C13	108.9 (3)	C30—C31—C33	107.7 (3)
C6—C12—C13	121.1 (3)	C26—C32—C33	122.2 (3)
C6—C12—C5	128.5 (3)	C26—C32—C25	127.4 (3)
C13—C12—C5	110.4 (3)	C33—C32—C25	110.4 (3)
C9—C13—C12	119.4 (3)	C32—C33—C29	118.6 (3)
C9—C13—C11	133.0 (3)	C32—C33—C31	108.4 (3)
C12—C13—C11	107.5 (3)	C29—C33—C31	133.0 (3)
O1—C14—C9	122.1 (3)	O2—C34—C29	122.0 (3)
O1—C14—C15	122.0 (3)	O2—C34—C35	121.9 (3)
C9—C14—C15	115.9 (3)	C29—C34—C35	116.1 (3)
C14—C15—Cl3	113.4 (2)	C34—C35—Cl6	113.8 (2)
C14—C15—H15A	108.9	C34—C35—H35A	108.8
Cl3—C15—H15A	108.9	Cl6—C35—H35A	108.8
C14—C15—H15B	108.9	C34—C35—H35B	108.8
Cl3—C15—H15B	108.9	Cl6—C35—H35B	108.8
H15A—C15—H15B	107.7	H35A—C35—H35B	107.7
			
C11—C1—C2—C3	0.3 (5)	C31—C21—C22—C23	0.9 (5)
C1—C2—C3—C4	1.3 (5)	C21—C22—C23—C24	−1.5 (6)
C1—C2—C3—Cl1	−178.6 (3)	C21—C22—C23—Cl4	177.8 (3)
C2—C3—C4—C10	−0.7 (5)	C22—C23—C24—C30	−0.3 (5)
Cl1—C3—C4—C10	179.1 (2)	Cl4—C23—C24—C30	−179.5 (3)
C12—C6—C7—C8	−1.4 (5)	C32—C26—C27—C28	0.5 (5)
C12—C6—C7—Cl2	179.1 (3)	C32—C26—C27—Cl5	−179.3 (3)
C6—C7—C8—C9	−0.9 (5)	C26—C27—C28—C29	−0.3 (5)
Cl2—C7—C8—C9	178.6 (3)	Cl5—C27—C28—C29	179.4 (3)
C7—C8—C9—C13	3.3 (5)	C27—C28—C29—C33	−1.0 (5)
C7—C8—C9—C14	−171.5 (3)	C27—C28—C29—C34	174.8 (3)
C3—C4—C10—C11	−1.5 (5)	C23—C24—C30—C31	2.5 (5)
C3—C4—C10—C5	177.6 (3)	C23—C24—C30—C25	−177.7 (3)
C12—C5—C10—C4	−179.6 (3)	C32—C25—C30—C24	−179.9 (3)
C12—C5—C10—C11	−0.4 (3)	C32—C25—C30—C31	−0.1 (4)
C2—C1—C11—C10	−2.5 (5)	C22—C21—C31—C30	1.2 (5)
C2—C1—C11—C13	−179.1 (3)	C22—C21—C31—C33	179.2 (3)
C4—C10—C11—C1	3.1 (5)	C24—C30—C31—C21	−3.0 (5)
C5—C10—C11—C1	−176.1 (3)	C25—C30—C31—C21	177.2 (3)
C4—C10—C11—C13	−179.6 (3)	C24—C30—C31—C33	178.5 (3)
C5—C10—C11—C13	1.2 (3)	C25—C30—C31—C33	−1.3 (4)
C7—C6—C12—C13	1.2 (5)	C27—C26—C32—C33	0.8 (5)
C7—C6—C12—C5	−179.0 (3)	C27—C26—C32—C25	−179.5 (3)
C10—C5—C12—C6	179.6 (3)	C30—C25—C32—C26	−178.2 (3)
C10—C5—C12—C13	−0.6 (3)	C30—C25—C32—C33	1.6 (4)
C8—C9—C13—C12	−3.4 (4)	C26—C32—C33—C29	−2.2 (5)
C14—C9—C13—C12	171.2 (3)	C25—C32—C33—C29	178.1 (3)
C8—C9—C13—C11	176.7 (3)	C26—C32—C33—C31	177.3 (3)
C14—C9—C13—C11	−8.8 (5)	C25—C32—C33—C31	−2.4 (4)
C6—C12—C13—C9	1.2 (5)	C28—C29—C33—C32	2.2 (5)
C5—C12—C13—C9	−178.6 (3)	C34—C29—C33—C32	−173.4 (3)
C6—C12—C13—C11	−178.9 (3)	C28—C29—C33—C31	−177.1 (3)
C5—C12—C13—C11	1.3 (3)	C34—C29—C33—C31	7.3 (6)
C1—C11—C13—C9	−4.8 (6)	C21—C31—C33—C32	−175.9 (4)
C10—C11—C13—C9	178.4 (3)	C30—C31—C33—C32	2.3 (4)
C1—C11—C13—C12	175.3 (3)	C21—C31—C33—C29	3.5 (6)
C10—C11—C13—C12	−1.6 (3)	C30—C31—C33—C29	−178.3 (3)
C13—C9—C14—O1	−40.8 (5)	C28—C29—C34—O2	−133.1 (4)
C8—C9—C14—O1	133.8 (4)	C33—C29—C34—O2	42.6 (5)
C13—C9—C14—C15	138.3 (3)	C28—C29—C34—C35	49.7 (4)
C8—C9—C14—C15	−47.1 (4)	C33—C29—C34—C35	−134.6 (3)
O1—C14—C15—Cl3	−8.4 (5)	O2—C34—C35—Cl6	31.8 (5)
C9—C14—C15—Cl3	172.5 (3)	C29—C34—C35—Cl6	−151.0 (3)

### Hydrogen-bond geometry (Å, °)

**Table:**

*D*—H···*A*	*D*—H	H···*A*	*D*···*A*	*D*—H···*A*
C1—H1···O1i	0.93	2.37	2.998 (4)	124
C15—H15B···Cl6i	0.97	2.80	3.678 (5)	151
C21—H21···O2i	0.93	2.45	3.086 (5)	126
C35—H35A···O1^i^	0.97	2.45	3.261 (5)	140

Symmetry codes: i; (i) *x*−1, *y*, *z*.
